# Excess all-cause mortality during the COVID-19 pandemic in Europe – preliminary pooled estimates from the EuroMOMO network, March to April 2020

**DOI:** 10.2807/1560-7917.ES.2020.25.26.2001214

**Published:** 2020-07-02

**Authors:** Lasse S Vestergaard, Jens Nielsen, Lukas Richter, Daniela Schmid, Natalia Bustos, Toon Braeye, Gleb Denissov, Tatjana Veideman, Oskari Luomala, Teemu Möttönen, Anne Fouillet, Céline Caserio-Schönemann, Matthias an der Heiden, Helmut Uphoff, Theodore Lytras, Kassiani Gkolfinopoulou, Anna Paldy, Lisa Domegan, Joan O'Donnell, Francesca de’ Donato, Fiammetta Noccioli, Patrick Hoffmann, Telma Velez, Kathleen England, Liselotte van Asten, Richard A White, Ragnhild Tønnessen, Susana P da Silva, Ana P Rodrigues, Amparo Larrauri, Concepción Delgado-Sanz, Ahmed Farah, Ilias Galanis, Christoph Junker, Damir Perisa, Mary Sinnathamby, Nick Andrews, Mark O'Doherty, Diogo FP Marquess, Sharon Kennedy, Sonja J Olsen, Richard Pebody, ECDC Public Health Emergency Team for COVID-19, Tyra G Krause, Kåre Mølbak, ECDC Public Health Emergency Team for COVID-19, ECDC Public Health Emergency Team for COVID-19, Pasi Penttinen, Nick Bundle, Cornelia Adlhoch

**Affiliations:** 1 Statens Serum Institut, Copenhagen, Denmark; 2 Austrian Agency for Health and Food Safety, Vienna, Austria; 3 Sciensano, Brussels, Belgium; 4 National Institute for Health Development, Tallinn, Estonia; 5 Finnish National Institute for Health and Welfare, Helsinki, Finland; 6 French Public Health Agency (Santé Publique France), Saint-Maurice, France; 7 Robert Koch Institute, Berlin, Germany; 8 Hessisches Landesprüfungs- und Untersuchungsamt im Gesundheitswesen, Dillenburg, Germany; 9 National Public Health Organization, Athens, Greece; 10 National Public Health Institute, Budapest, Hungary; 11 Health Service Executive - Health Protection Surveillance Centre, Dublin, Ireland; 12 European Programme for Intervention Epidemiology Training (EPIET), European Centre for Disease Prevention and Control (ECDC), Stockholm, Sweden.; 13 Dipartimento Epidemiologia del S.S.R., Lazio - ASL Roma 1, Rome, Italy; 14 Health Directorate Luxembourg – Division de l’inspection sanitaire, Luxembourg; 15 Directorate for Health Information and Research, Ministry for Health, Malta; 16 National Institute for Public Health and the Environment (RIVM), Bilthoven, The Netherlands; 17 Norwegian Institute of Public Health, Oslo, Norway; 18 Instituto Nacional de Saúde Doutor Ricardo Jorge, Lisbon, Portugal; 19 National Centre of Epidemiology, CIBER Epidemiología y Salud Pública (CIBERESP), Carlos III Health Institute, Madrid, Spain; 20 Public Health Agency of Sweden, Stockholm, Sweden; 21 Federal Statistical Office, Neuchâtel, Switzerland; 22 Federal Office of Public Health, Bern, Switzerland; 23 Public Health England, Colindale, United Kingdom; 24 Public Health Agency, Northern Ireland, United Kingdom; 25 Public Health Scotland, Glasgow, United Kingdom; 26 World Health Organization, Regional Office for Europe, Copenhagen, Denmark; 27 The members of the ECDC Public Health Emergency Team for COVID-19 are listed below; 28 Department of Veterinary and Animal Science, Faculty of Health and Medical Science, University of Copenhagen, Copenhagen, Denmark

**Keywords:** All-cause mortality, Covid-19 pandemic, Europe, EuroMOMO

## Abstract

A remarkable excess mortality has coincided with the COVID-19 pandemic in Europe. We present preliminary pooled estimates of all-cause mortality for 24 European countries/federal states participating in the European monitoring of excess mortality for public health action (EuroMOMO) network, for the period March–April 2020. Excess mortality particularly affected  ≥ 65 year olds (91% of all excess deaths), but also 45–64 (8%) and 15–44 year olds (1%). No excess mortality was observed in 0–14 year olds.

We present pooled European-wide weekly mortality estimates from the European monitoring of excess mortality for public health action (EuroMOMO) network from the beginning of 2020 until week 18 (23 April–3 May) of this year. This period includes the initial 2 months of the coronavirus disease (COVID-19) pandemic in Europe, March and April, a time frame characterised by the end of the influenza season but widespread COVID-19 community transmission. We also calculate the weekly and cumulative excess all-cause mortality from week 1 to week 18/2020, and compare the results to the same period of the previous 4 years (2016, 2017, 2018, and 2019).

## Relevance of excess mortality monitoring during the coronavirus disease pandemic in Europe 

Following a coronavirus disease (COVID-19) outbreak in China in late December 2019, the causative virus, severe acute respiratory syndrome coronavirus 2 (SARS-CoV-2), spread rapidly to become a major global public health emergency [1,2]. On 11 March 2020, COVID-19 was declared a pandemic [3], which is currently still ongoing. In Europe, the first COVID-19 cases were reported in January 2020 in France [4]. During the following weeks, occurrences of cases and fatalities with rapidly increasing numbers were observed across many European countries [5,6]. By the end of June 2020 [7], about 1.6 million confirmed COVID-19 cases and 177,000 deaths had been officially reported from European Union (EU)/European Economic Area (EEA) countries and the United Kingdom (UK). 

The official national statistics on COVID-19 cases and deaths among European countries are heterogeneous, partly due to the differences in applied testing strategies and access to testing, and use of different reporting modalities. In this situation, numbers of excess all-cause deaths can provide a more complete and timely proxy measure of the mortality burden of COVID-19 in the population, in particular when there are no other factors known to cause excess mortality, such as seasonal influenza [8].

Since 2009, following the influenza A(H1N1)pdm09 pandemic, the EuroMOMO network (www.euromomo.eu) has monitored the weekly all-cause excess mortality in a large number of countries across Europe. EuroMOMO uses a statistical algorithm, which allows to compare and pool national mortality estimates [9]. The EuroMOMO mortality outputs form part of the routine monitoring of seasonal influenza severity in Europe, producing weekly and end-of-season reports to inform national and international public health agencies, and to evaluate mortality signals within and between countries in a systematic and timely manner [10-12]. Such outputs are particularly useful in the context of an emerging pandemic caused by a new infectious agent, where the true mortality burden is difficult to ascertain and compare between countries.

## Estimating the number of all-cause deaths in EuroMOMO countries

Countries participating in the EuroMOMO network collect weekly data from civil registers or other official reporting sources on the number of deaths of all causes. The all-cause excess mortality, defined as the observed minus the expected numbers of deaths, is estimated using the EuroMOMO statistical algorithm, previously described in detail [9]. The EuroMOMO hub compiles these weekly data from individual countries and conducts a pooled analysis using an age-stratified method [13].

Currently, the following 24 European countries or federal states participate with their weekly data submission: Austria, Belgium, Denmark, England (UK), Estonia, Finland, France, Germany (Berlin and Hesse), Greece, Hungary, Ireland, Italy (19 cities), Luxembourg, Malta, the Netherlands, Northern Ireland (UK), Norway, Portugal, Scotland (UK), Spain, Sweden, Switzerland and Wales (UK). Ireland has encountered additional delays in death registrations during the pandemic period, hence the included numbers for this country are not yet complete.

We present preliminary pooled European-wide mortality estimates from the EuroMOMO network for 2020. The pooled estimates cover the period until the end of week 18 (3 May)/2020, based on data received by the end of week 23 (7 June) of this year. Estimates are shown for all ages combined, and by the age groups 0–14, 15–44, 45–64, 65–74, 75–84, and ≥ 85 years. In addition to weekly all-cause mortality estimates, we also calculate the weekly and cumulative excess all-cause mortality for 2020 up to week 18, and compare the results with the same period in each of the previous 4 years (2016, 2017, 2018, and 2019) using our standard approach.

Due to delay in death registration, the data for the most recent weeks beyond week 18 2020 are not included in the present report, but are available from the EuroMOMO website, where estimates corrected for delay in registration using a country-specific adjustment function are shown. 

## Ethical statement

Ethical approval was not needed for the study, which is based on surveillance data only.

## Pooled estimates of all-cause excess mortality

All-cause mortality started to exceed normal expected levels in Italy around week 10 (1–8 March)/2020. In the following weeks, excess mortality was also detected in several other EuroMOMO countries, including the following: Belgium, England (UK), France, the Netherlands, Northern Ireland (UK), Portugal, Scotland (UK), Spain, Sweden, Switzerland and Wales (UK). While, during the same period of the COVID-19 pandemic, several other countries experienced no or only very limited excess mortality including: Austria, Denmark, Estonia, Finland, Germany (Berlin and Hesse federal states), Greece, Hungary, Luxembourg, Malta and Norway.

**Figure 1:**
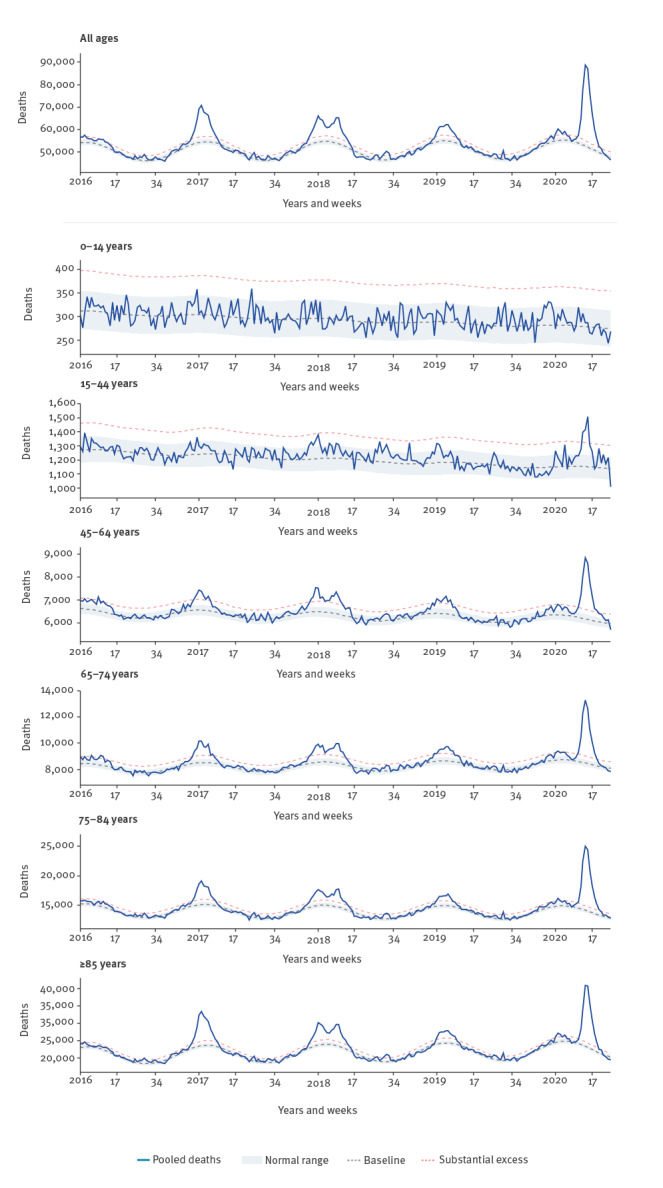
EuroMOMO pooled estimates of all-cause mortality shown for all ages combined and by age group, week 1/2016−week 18/2020

The pooled mortality estimates for the 24 participating European countries or federal states showed an increasing trend during the first weeks of March 2020, and an excess mortality level higher than four z-scores above the baseline (defined as ‘substantial excess’) in week 11 (9–15 March)/2020 (Figure 1). The mortality was highest among individuals aged 65 years and older, but some countries also observed marked excess deaths among those aged 45–64 years, and some countries (in particular England and Spain) even noted excess mortality in the age group 15–44 years, also reflected in the overall pooled estimates. No excess mortality was observed in children aged 0–14 years.

EuroMOMO: European monitoring of excess mortality for public health action.

Substantial excess mortality is defined as an excess level equivalent to four z-scores above the expected baseline.

Mortality increased steeply in the next 3 weeks and peaked in all countries during week 14 (30 March–5 April)/2020, when a total of 88,581 deaths (all ages) was reached, translating into a z-score of 58. By week 15 (6–12 April)/2020 the mortality started a rapid decline, affecting all age groups except the 0–14 years where no excess mortality had been observed; however, by week 18/2020 a substantial mortality for all ages combined, of around 60,000 deaths, was still seen, corresponding to a z-score of 16 above the baseline.

**Figure 2:**
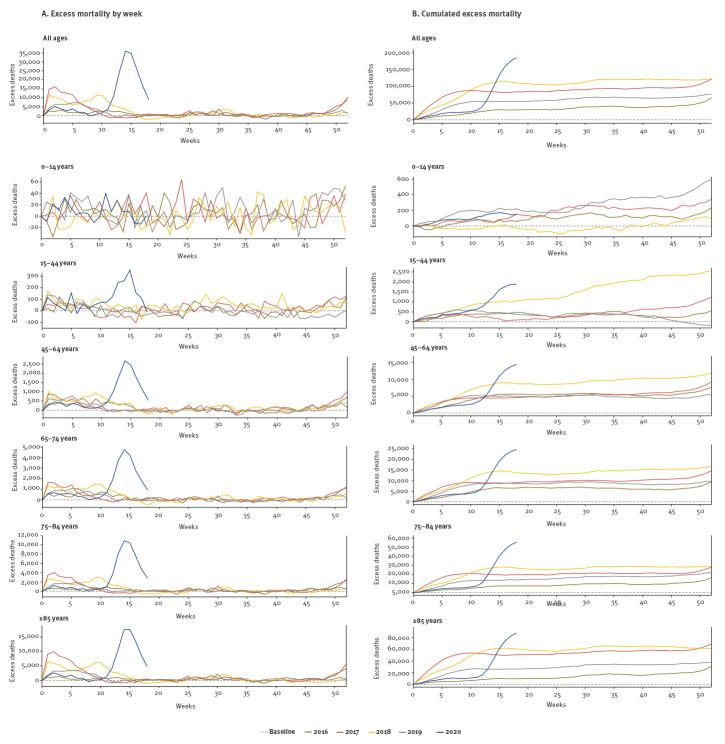
EuroMOMO pooled estimates of excess^a^ all-cause mortality shown combined for all ages and by age group, from week 1 to week 18 for year 2020, and week 1 to week 52 for the years 2016, 2017, 2018, 2019, respectively

Figure 2 shows the weekly and cumulative pooled excess all-cause mortality estimates observed during the COVID-19 pandemic in comparison to the previous 4 years, from week 1 to week 18. At the peak level of mortality, in week 14, an excess of 35,802 deaths across all ages was estimated, of which 32,815 (92%) were persons aged ≥ 65 years. In comparison, the highest excess mortality in any week during the previous 4 years reached 16,165 deaths (all ages) in week 2 in 2017, i.e. during the severe 2016/17 influenza season [11] (Figure 2A).

EuroMOMO: European monitoring of excess mortality for public health action.

^a^ Excess is defined as >2 z scores.

The cumulative excess mortality from week 1 to week 18/2020 reached a total of 185,287 deaths (all ages), including 24,438 (13%) in persons aged 65–74 years, 55,226 (30%) in persons aged 75–84 years, and 88,598 (48%) in persons aged ≥ 85 years. The cumulative deaths in the younger age groups reached 14,339 (8%) in 45–64 year-old persons and 1,843 (1%) in 15–44 year-old persons. This period of the year includes a part of the usual influenza season. In comparison, the cumulative excess deaths (all ages) by week 18 reached 55,441 in 2019, 110,483 deaths in 2018, 83,009 deaths in 2017 and 29,849 deaths in 2016 (Figure 2B).

## Discussion

Soon after its detection in China in late 2019, COVID-19 was found to lead to a considerable morbidity and mortality burden. A systematic review and meta-analysis resulted in an overall estimated proportion of severe cases of 25.6% and a case fatality rate (CFR) of 3.6%, with more severe clinical symptoms and higher CRF among older patients and patients with underlying medical conditions [14]. As increasing age and comorbidity appear to pose a risk for fatal outcome, it can be argued that COVID-19 mainly leads to death in patients with an expected short life span so that the overall excess mortality at the population level may be relatively limited. However, our analysis suggests that transmission of COVID-19 indeed has had a marked impact on all-cause mortality in the European population, despite the extensive societal preventive measures taken and the increase of treatment capacity in affected countries. We observed steep peaks in excess mortality in the age groups 65–74 years, 75–84 years and ≥ 85 years, respectively, considerably exceeding the excess mortality levels observed during any of the past influenza seasons monitored by EuroMOMO. Excess mortality was also observed in persons aged 45–64 years and 15–44 years, although to a much smaller extent than was seen among elderly people. No excess mortality was observed in children under 15 years old.

When facing a new viral pandemic such as COVID-19, with many unknowns regarding biology and transmission potential, estimating the impact on public health in terms of disease severity and mortality is critical. With limited testing capacity, changing testing strategies and different surveillance and reporting systems, the officially reported mortality statistics based on individual COVID-19 death reports will inevitably be heterogeneous and incomplete. In this situation, estimating excess all-cause mortality using a standard approach across countries provides a powerful tool to rapidly obtain unbiased estimates of the COVID-19 mortality burden, and how it affects different age groups and different countries and areas. The mortality impact of the COVID-19 epidemic was clearly demonstrated by reports of the excess all-cause mortality estimates by the Ministry of Health in Italy in March 2020 [15,16], and by weekly all-cause mortality reports published early in the epidemic by the national health authorities of several other European countries.

All-cause excess mortality is estimated in the current study. Considering the limited occurrence of seasonal influenza during the peak time of the COVID-19 mortality in the participating countries, and the absence of other major public health events, the estimated excess mortality can primarily be attributed to COVID-19. Some of these deaths may be directly related to COVID-19; others indirectly due to delays in accessing healthcare for other illnesses, and others due to other factors. The COVID-19 pandemic in Europe is not over yet, and in the coming weeks and months, as the national mortality data become more complete, more definitive estimates of the mortality burden of COVID-19 in Europe will be available and comparisons to previous influenza epidemics/pandemics and other public health events can be made. Similarly, observed discrepancies between all-cause mortality estimates and officially notified mortality statistics can be evaluated, to guide future COVID-19 case reporting and surveillance efforts.

In the current COVID-19 pandemic situation, the EuroMOMO system has proven to be a valuable tool for timely detection and reporting of excess all-cause mortality across many parts of Europe in a coordinated and consistent manner. National and international organisations, the general public, media and others have largely drawn on EuroMOMO as a source of timely and easily accessible information about the evolving pandemic. The EuroMOMO network welcomes any country within Europe to become part of the network and thereby contribute to an even wider geographical coverage of the ongoing monitoring of the COVID-19 pandemic, from which new waves of transmission could occur. Importantly, the EuroMOMO statistical algorithm applied at the national level data provides countries with a simple and easy-to-use national mortality monitoring system. These mortality data are crucial for early warning and impact assessment, informing policy decisions and public health action.

## Note

The members of the ECDC Public Health Emergency Team for COVID-19 are affiliated with the European Centre for Disease Prevention and Control, Solna, Sweden.
